# How Do Australian Health Professionals Working in a Rural Emergency Department Perceive Their Work With People With Mental Illness? An Interview Study

**DOI:** 10.1111/inm.70293

**Published:** 2026-07-02

**Authors:** Peter Nathan, Niels Buus, Alison Hansen

**Affiliations:** ^1^ School of Nursing and Midwifery Monash University Melbourne Australia; ^2^ South West Hospital and Health Service Queensland Health Roma Australia; ^3^ Department of Public Health Aarhus University Aarhus Denmark

**Keywords:** health professionals, mental illness, reflexive thematic analysis, relational safety, rural emergency departments, rural nursing, stigma

## Abstract

Emergency departments (EDs) are often the first point of contact for individuals in mental health crisis. In rural Australia, health professionals face distinctive challenges shaped by limited resources, workforce shortages and overlapping professional and community roles. These conditions influence how health professionals perceive, engage with and respond to people experiencing mental health crisis. To explore how health professionals working in a rural emergency department perceive their work with people presenting with mental illness. A qualitative design using semi‐structured interviews was employed with nine participants (six nurses and three medical officers) from a rural ED in South West Queensland. Data were analysed using Braun and Clarke's Reflexive Thematic Analysis. Three interrelated themes captured participants' perceptions: safety as relational and negotiated practice, education and training as relational and experiential learning and small‐town mentality. Findings suggested that care in rural EDs is sustained less by systems and protocols than by human connection, local knowledge and moral endurance. Participants described navigating safety through empathy and teamwork, constructing learning through shared experience rather than formal training and managing the tensions of visibility, stigma and relationships in close‐knit communities. Work with people with mental health illness in rural emergency settings is perceived as relational, adaptive and context bound. Building sustainable rural mental health responses may benefit from embedding specialist support, accessible education and reflective spaces that honour both professional wellbeing and community interdependence.

## 1 Introduction

Emergency departments worldwide are experiencing rising numbers of mental‐health presentations, reflecting a broader international trend in acute mental‐health service utilisation (World Health Organization 2022). This pattern is mirrored across Australia, where emergency departments recorded more than 310 000 mental health–related presentations between 2023 and 2024 (AIHW 2024), with national data showing a 55 262‐case increase over the past decade (AIHW 2025). International research highlights that emergency clinicians frequently encounter clinical, emotional and environmental challenges when responding to acute mental health crises, with similar concerns reported in the United States, Canada, Brazil, and Australia (McIntosh 2021). While these challenges are recognised across multiple health systems, they are intensified in Australian rural emergency departments, where geographic isolation, limited specialist availability and reliance on generalist staff create distinct conditions for care. These structural inequities mean that rural clinicians often manage complex psychiatric presentations with fewer resources and reduced access to mental health specialists. As mental health presentations increase, emergency clinicians must make rapid decisions about triage, safety and resource allocation within time‐pressured environments. In rural settings, such decisions are shaped not only by protocols but also by clinicians' judgements and interpretations of risk, responsibility and care. Understanding how rural clinicians perceive and navigate these pressures is therefore analytically important for explaining how mental health care is enacted in practice.

While the complexity of mental health presentations in EDs has been widely documented (McIntosh 2021; Peart et al. 2023), much less attention has been given to how these experiences are understood and managed within rural contexts, where resource scarcity and community familiarity shape care in distinctive ways.

Rural EDs face systemic challenges that extend beyond clinical workload. Health professionals frequently practice with minimal psychiatric support and a workforce dominated by generalist practitioners. Although telehealth services have expanded in recent years, access to after‐hours specialist mental health support in rural and remote regions remains geographically variable and often limited (AIHW 2024; National Rural Health Alliance 2023; Beks et al. 2018). While the prevalence of mental illness in rural Australia mirrors that of metropolitan regions, access to specialist care remains significantly reduced (National Rural Health Alliance 2021). As a result, ED clinicians often assume responsibility for complex psychiatric presentations in environments not designed for sustained mental health care.

These structural limitations shape how health professionals make decisions about safety and engagement. Limitations in psychiatric support, after‐hours services and workforce specialisation shape how health professionals make decisions about safety and engagement. In small rural communities, nurses and doctors may know patients outside the hospital, which can complicate professional boundaries and confidentiality. Privacy concerns and stigma may operate not only at a systemic level but also through social familiarity (Reed and Fitzgerald 2005). These contextual factors contribute to the complexity of rural emergency practice.

How health professionals perceive and engage with individuals diagnosed with mental illness has direct implications for safety, equity and recovery‐oriented care. Perceptions are shaped by experience, organisational culture and the values embedded in professional training (Isbell et al. 2020). In emergency care, these perceptions can determine triage decisions, communication tone and willingness to maintain therapeutic engagement. Studies across urban settings have found that ED clinicians often describe mental health presentations as more challenging and less rewarding than physical cases, citing unpredictability, limited time and emotional exhaustion (McIntosh 2021; Wong et al. 2016). In rural contexts, these perceptions may be compounded by professional isolation and the absence of formal support systems.

Studies on relational care emphasise that compassion and safety are co‐constructed within interactions rather than dictated by policy (Braithwaite et al. 2015). Within rural EDs, relationality takes on heightened significance: teamwork, empathy and improvisation become essential to maintaining both person and staff safety (Pawaskar et al. 2022). These tensions suggest that safety is experienced as a negotiated process shaped by contextual and systemic factors. Furthermore, nurses and doctors describe moments of moral distress when systemic limitations prevent them from delivering the care they believe is ethically right (Zavotsky and Chan 2016).

Workforce education remains a persistent gap in rural health systems. Previous research identifies inadequate mental health training among emergency clinicians as a key barrier to effective practice (Peart et al. 2023). For rural staff, opportunities for structured education are often sporadic or dependent on distance learning, leaving many to rely on experiential and peer‐based learning (Beks et al. 2018). Understanding how clinicians conceptualise and construct learning within this context is critical for designing sustainable educational interventions that reflect rural realities rather than metropolitan assumptions.

Cultural proximity is a defining feature of rural life. Health professionals frequently encounter patients who are neighbours, acquaintances or relatives of colleagues, complicating notions of confidentiality and professional distance. In such contexts, stigma becomes both interpersonal and communal, a shared social phenomenon that affects how mental illness is seen and spoken about (Thornicroft et al. 2007). These dynamics challenge standardised models of care and call for a nuanced exploration of how community norms intersect with clinical practice in rural emergency settings. Within rural emergency departments, they shape how clinicians manage safety, negotiate professional boundaries and engage therapeutically with individuals experiencing mental health crisis.

Despite growing recognition of mental health inequity in rural Australia, few studies have examined how emergency clinicians themselves make sense of their experiences and responsibilities. Most existing work focuses on workforce shortages, policy frameworks or service outcomes, overlooking the lived professional realities that shape decision‐making and empathy in situ (Pawaskar et al. 2022). This study therefore centres the perceptions of rural ED nurses and doctors, exploring how they perceive and engage with people presenting with mental illness within the relational, systemic and cultural constraints of their environment.

To better understand these dynamics, concepts such as relational safety, moral distress and negotiated care provide an important lens for examining how clinicians navigate mental health presentations in emergency settings. These ideas inform the conceptual orientation of this study by highlighting the relational, ethical and contextual dimensions of clinical decision‐making in rural emergency departments. Attending to these dynamics supports analysis of how clinicians perceive and respond to mental health presentations within resource‐constrained environments.

The aim of this study was to explore how Australian health professionals working in a rural emergency department perceive their work with people diagnosed with a mental illness. By examining their experiences, attitudes and challenges, the research seeks to inform contextually grounded workforce education, policy reform and support structures that promote compassionate, safe and recovery‐oriented care in rural emergency settings.

## 2 Methods

### 2.1 Study Design

This study adopted an interpretivist qualitative design to explore how health professionals in a rural ED perceive their work with people with a mental illness. The interpretivist paradigm recognises that knowledge is socially constructed and shaped through context, interaction and professional experience (Creswell and Poth 2018). This worldview supported the use of Reflexive Thematic Analysis (RTA) as described by Braun and Clarke (2021), enabling a contextual interpretation of meaning rather than the pursuit of consensus or saturation.

### 2.2 Setting

The study was conducted in a rural emergency department in South West Queensland, Australia. The hospital serves as a referral hub for several surrounding rural communities with a combined population of approximately 13 000 people. The emergency department manages a range of presentations, including acute medical conditions and mental health crises and is staffed by registered nurses and medical officers providing 24‐h emergency care. Specialist mental health services in the region are limited, with a single community mental health service providing support primarily during standard business hours (Queensland Health 2024). The area is classified as Modified Monash Model category 4 (MMM4) indicating a medium‐sized rural community with limited access to specialist health services (Australian Government Department of Health and Aged Care 2023).

### 2.3 Participants and Recruitment

Participants were recruited using purposive sampling to ensure inclusion of clinicians with direct experience managing mental health presentations in the emergency department. Recruitment information was displayed within the ED, inviting interested staff to contact the first author directly. Inclusion criteria required a minimum of 1 year of clinical experience and direct involvement in caring for people presenting with mental health concerns. The sampling approach sought diversity of professional perspectives by including both registered nurses and medical officers with varying levels of clinical experience. To minimise potential power imbalances, clinicians with whom the first author had direct clinical or supervisory relationships were not eligible to participate. Recruitment continued until a sufficient range of perspectives relevant to the research question had been obtained, supporting analytic depth consistent with reflexive thematic analysis.

The first author's professional background and relationship to the study setting are also relevant to understanding the research process. The first author is a registered mental health nurse employed within the same health service but not within the emergency department where the study took place. This position provided familiarity with the rural health system and the organisational context in which participants worked while maintaining sufficient professional distance from the ED team. The first author's experience in rural mental health practice supported rapport during interviews and sensitivity to participants' descriptions of clinical challenges. Throughout the research process, reflexive awareness of this insider positioning was maintained through reflective journaling and discussion with the research team to ensure interpretations remained grounded in participants' accounts.

### 2.4 Data Collection

Data were collected through semi‐structured, one‐on‐one interviews conducted by the first author. Interviews were scheduled at times convenient to participants, including before or after shifts or on rostered days off. Each interview lasted between 45 and 60 min and took place in a private setting away from the clinical area to minimise distractions and ensure confidentiality. Semi‐structured interviews were chosen to allow participants the flexibility to share their experiences in their own words, while still ensuring consistency across key areas of inquiry (Brinkmann and Kvale 2015). This approach enabled the researcher to explore sensitive and context‐specific perspectives, particularly in relation to mental health presentations in rural emergency settings. A conversational structure also supported rapport building, which was essential for eliciting rich, in‐depth responses from participants reflecting on complex and sometimes emotionally charged aspects of their clinical practice (Brinkmann and Kvale 2015).

The interview guide (Appendix S1) was developed to explore clinicians' experiences of caring for people presenting with mental illness in a rural emergency department. The guide was informed by existing literature on emergency mental health care and refined through discussion in the research team to ensure alignment with the study aim. It was designed with an awareness about both the thematic content of the interview and the interpersonal dynamics throughout the interview, drawing on principles described by Brinkmann and Kvale (2015). Appendix S1 contains the semi‐structured interview guide used in this study.

### 2.5 Data Analysis

Reflexive Thematic Analysis (RTA) was used to analyse the data, guided by Braun and Clarke's (2021) six‐phase approach: (1) familiarisation, (2) generating initial codes, (3) constructing themes, (4) reviewing themes, (5) defining and naming themes and (6) producing the report. This approach provided a flexible yet rigorous framework for exploring participants' experiences and identifying patterns of meaning across the dataset. The analysis began with repeated readings of transcripts and audio recordings to ensure immersion in the data. Initial codes were manually generated to highlight segments reflecting participants' views, challenges and experiences in caring for people with mental illness in the ED. These codes were clustered into preliminary themes by the first author that captured both commonalities and differences across the data set and discussed with the team. These discussions involved reviewing coded extracts, questioning preliminary interpretations and refining theme boundaries to ensure analytic coherence and alignment with participants' accounts. Themes were iteratively refined as a team to ensure they remained grounded in the participants' language and context and were then clearly defined and named to reflect the lived realities described in the interviews. Theme development involved an iterative and interpretive process in which the first author actively examined patterns of meaning across participants' accounts. Rather than simply grouping similar codes, the analysis sought to interpret how clinicians made sense of their experiences within the rural emergency context. Reflexive engagement with the data allowed themes to move beyond descriptive categories towards interpretive insights about relational safety, experiential learning and community dynamics in rural emergency care.

### 2.6 Rigour and Trustworthiness

Rigour and trustworthiness were supported through transparency, reflexivity and coherence, consistent with Braun and Clarke's (2021) reflexive thematic analysis. Quality in this study was understood not as the replication of findings but as the clarity and transparency of the analytic process. Reflexivity was central throughout; the researcher maintained a reflexive journal documenting assumptions, emerging interpretations and analytical decisions, which supported ongoing reflection about how professional experience in rural mental health practice might shape interpretation of the data. The research team met regularly to discuss emerging interpretations and review coded extracts, helping refine theme boundaries and question preliminary assumptions. Rich, contextualised participant quotations were used to demonstrate credibility and ensure that themes remained grounded in participants' accounts. A clear audit trail of coding, theme development and refinements was maintained to document analytic decisions throughout the study, strengthening dependability and confirmability and contributing to the overall trustworthiness of the research.

### 2.7 Ethics

This study received ethical approval from the Darling Downs Health Human Research Ethics Committee (DDHREC/2025/000) and Monash University Human Research Ethics Committee (MUHREC). Participants were informed that participation was voluntary and that they could withdraw at any time without consequences. Verbal and written consent was obtained prior to interviews with assurance that their responses would remain confidential and de‐identified in all reporting. Transcripts were anonymised and a pseudonym assigned to preserve confidentiality. Participants were given the opportunity to review and clarify their transcripts prior to analysis; however, none chose to do so. Data were securely stored on a password protected institutional network provided by Monash University.

## 3 Findings

Participants included nine health professionals, six registered nurses and three medical officers. Data generated three interrelated themes that together form a coherent set of findings capturing how rural ED health professionals perceive their work with people presenting with mental illness. The themes of safety as relational and negotiated practice, education and training as relational and experiential learning and a third, small town mentality illustrate a complex interplay between systemic, interpersonal and cultural factors. Each theme is described below and supported by key quotes to preserve the authenticity of participants' perceptions.

### 3.1 Safety as a Relational and Negotiated Practice

Participants frequently described safety in the rural emergency department as uncertain and emotionally demanding, particularly when clinicians managed aggressive behaviour without security support or specialised mental health services. As Jane, a nurse (Participant #9), explained when recalling an escalating encounter with a patient:

I was scared for our personal safety. She kept coming into the nurses' station and was standing in the entrance. She was verbally threatening towards us, so scare. She was unable to be de‐escalated; she was very angry. The doctor was on the phone with the acute care team who basically said she'd be fine for discharged, and she really just stormed out and I don't know, in hindsight if that was the safest thing, but for us it was probably the safest thing. (Participant #9)

Jane's account illustrates the uncertainty clinicians described when negotiating safety decisions in situations when structural supports was limited. Similar concerns were expressed by several participants, who described feeling vulnerable when managing aggression without security support or specialist mental health staff. Across participants' accounts, safety was described not as a fixed conditions ensured by institutional systems, but as a constant negotiation shaped by the immediate context of rural emergency practice. For some clinicians, this negotiation occurred within small teams where staff were required to respond to escalating situations without access to security personnel or designated safe spaces within the department.

Participants described situations in which verbally aggressive behaviour created a heightened sense of vulnerability, particularly when staff worked in small teams during evenings or weekends. In these moments, participants described balancing concern for their own safety with their professional responsibility to provide care. Discharging the individual sometimes felt like the safest immediate option for staff, yet participants reflected on the ethical tension this created, recognising that safety for staff could also mean reduced support for the person in crisis. In the absence of clear institutional safeguards, participants described relying on relational approaches to maintain safety. Strategies such as calm communication, empathy and responding to what mattered most to the person were frequently used to de‐escalate tension. These accounts suggest that safety in the rural ED was often maintained through interpersonal skill and teamwork rather than through physical infrastructure or formal protocols.

Participants also highlighted the broader structural limitations that shaped these safety negotiations. Limited access to specialist mental health services and delays in transferring individuals under involuntary orders meant that some people remained in the ED for extended periods. During these times, clinicians described relying on police presence as a form of proxy security, reinforcing participants' accounts that safety was often sustained through improvisation rather than formal system design. Across the accounts, safety emerged as a relational practice shaped by institutional constraints and the interpersonal strategies clinicians used to manage risk. Nurses often emphasised teamwork, empathy and mutual protection when managing safety, while medical officers more frequently referred to procedural responsibilities and system accountability. Together, these accounts suggest that participants experienced safety as a shared negotiation between human presence and limited structural support, sustained by clinicians who balance compassion with responsibility in resource‐constrained rural emergency settings.

### 3.2 Education and Training as Relational and Experiential Learning

Participants frequently described the absence of structured mental health education within the rural emergency department. As Maree, a nurse (Participant #3), reflected when discussing training opportunities for staff:

Well, there's no extra training and education. There's no in‐service that are provided. There's no extra courses apart from trauma that are provided. I think more awareness about the mental health programs would be beneficial. New staff aren't actually offered any mental health training. I feel that not many nursing staff know much about different diagnosis, and people know about palliative care and continence and diabetes, but mental health education isn't provided. (Participant #3)

Maree's reflection illustrates participants' broader concern about the limited availability of structured mental health education within the rural emergency department. In response to this limited availability of formal education, other participants described relying on experiential learning, observation and informal discussion with colleagues rather than structured training.

In the absence of regular in‐services or specialist mental health educators, participants described relying on exposure, observation and repetition to build confidence in managing mental health presentations. Many participants spoke about developing skills through day‐to‐day encounters with patients, learning from colleagues and reflecting on difficult situations after the event. Without consistent access to formal education or on‐site mentorship, clinicians often worked in small teams where opportunities for cross‐disciplinary learning were limited. In this context, confidence was frequently built through experience rather than through structured guidance.

Participants described learning as something they assembled for themselves, often under pressure and without a clear framework to guide practice. Rather than formalised training pathways, clinicians relied on informal conversations with senior colleagues, shared problem‐solving within the team and personal reflection following challenging encounters. Participants contrasted their rural learning environment with metropolitan emergency departments, where mental health educators or consultation‐liaison teams may provide immediate guidance and structured teaching. Participants described rural learning environments as largely reactive. Knowledge was often constructed after the event, through discussion and reflection within the team.

Despite these limitations, participants also described strengths emerging from this relational approach to learning. Informal teaching through teamwork, observation and shared experience contributed to a form of practical knowledge that was closely connected to the realities of rural emergency practice. Across participants, nurses tended to describe learning as relational and grounded in shared experience, whereas medical officers tended to emphasise system‐level coordination and cross‐workforce education. Taken together, these perspectives suggest that learning within rural emergency care was sustained less through formal educational programs and more through collective effort and adaptation shaped by local context.

### 3.3 Small Town Mentality

Participants frequently described how familiarity within small rural communities shaped care in the emergency department. The term small‐town mentality refers to the social dynamics of rural communities where familiarity, visibility and overlapping personal and professional relationships shape how mental health care is experienced and delivered. As Anne, a nurse (Participant #1), explained when reflecting on privacy and stigma:

I suppose with our mental health patients, there can be a bit of a stigma of shame. We want to give them the opportunity that they don't feel like everyone's looking at them and talking about them behind their backs. I suppose it's nice to kind of do a little bit extra just to facilitate that privacy, not have them spotted by people that they would see down the street. (Participant #1)

Anne's reflection illustrates participants' awareness of the social visibility experienced by people presenting with mental illness in small rural communities. Across participants' accounts, clinicians described taking deliberate steps to protect privacy and reduce stigma when caring for people known within the community.

In the rural emergency department, care was often inseparable from community life. Staff and people receiving care frequently knew one another beyond the hospital setting, creating a context where familiarity, empathy and stigma coexisted. Participants described practical actions taken to reduce visibility and protect privacy, such as closing curtains, offering quieter spaces within the department or adjusting communication to minimise attention from others in the waiting area. These gestures reflected an awareness that maintaining dignity was particularly important in communities where anonymity was limited.

At the same time, participants spoke about the emotional complexity of repeated encounters with individuals known within the community. Familiarity sometimes fostered compassion and continuity of care, yet it could also contribute to fatigue when clinicians regularly cared for the same individuals presenting in crisis. Participants also described situations where professional boundaries were blurred through dual relationships. Treating acquaintances, neighbours or colleagues created additional ethical considerations, as clinicians balanced professional responsibility with social familiarity. Across the accounts, nurses and medical officers described these dynamics differently. Nurses frequently described the emotional labour involved in protecting privacy and countering community stigma. Medical officers, meanwhile, often discussed the challenge of maintaining professional boundaries in communities where relationships extended beyond the hospital. These accounts illustrate how participants experienced rural emergency care as shaped by social proximity and community familiarity. Within small communities, therapeutic practice involves not only managing illness but also navigating the social realities of belonging, visibility and trust.

## 4 Discussion

This study explored how rural ED health professionals perceive and engage with people diagnosed with a mental illness. Through Braun and Clarke's (2021) Reflexive Thematic Analysis, three interrelated themes were developed: safety as relational and negotiated practice, education and training as relational and experiential learning and small‐town mentality. Together, these findings illuminated how care in rural EDs is constrained by structural limitations, with clinicians often assuming responsibility for managing safety and care in the absence of adequate system support.

In the first theme, participants' accounts revealed that safety in the rural ED is less about institutional control and more about ethical negotiation. Consistent with prior research (Isbell et al. 2020; Pawaskar et al. 2022), health professionals described maintaining safety through empathy, calm communication and relational presence rather than formal policy. However, in this study, safety also appeared to be shaped by considerations of gender and moral responsibility, with some participants describing how working in small, predominantly female teams influenced how they negotiated the balance between compassion and self‐protection when police or security support were unavailable.

The analysis suggested that health professionals felt that they often remained present despite fear and uncertainty. In the absence of formal safeguards, safety was described as a shared ethical responsibility sustained through trust, empathy and professional commitment.

These findings reinforce earlier literature describing relational safety as a co‐constructed process, as reflected in participants' reliance on empathy, communication and teamwork to manage risk in the absence of structural support. They also align with Zavotsky and Chan's (2016) account of moral distress in emergency nursing, with participants describing tensions between maintaining personal safety and fulfilling their duty of care. Yet in this rural context, such distress appeared amplified by isolation and limited back‐up, underscoring the need for dedicated support structures—such as debriefing, supervision and specialist liaison roles—to sustain health professionals' wellbeing and ethical resilience.

The second theme highlighted how rural ED health professionals construct learning as an informal, relational practice rather than a formal system. Participants' reliance on observation and experience reflects the chronic under‐provision of mental health education in rural emergency settings (Peart et al. 2023; Reed and Fitzgerald 2005). The absence of structured education produced both vulnerability and ongoing demands for adaptation, with clinicians often feeling underprepared while developing skills through necessity rather than structured support.

Interpreted reflexively, this pattern suggests that experiential learning has become normalised within rural emergency care, functioning as a pragmatic response to limited access to formal mental‐health education and specialist support. This finding supports Beks et al. (2018), who reported that rural nurses often rely on experiential, on‐the‐job learning when mental health specialists are unavailable. However, the present study adds a relational dimension: learning occurred through trust, dialogue and reflection within the team, with clinicians developing competence in the absence of consistent formal support. Such “relational apprenticeship” aligns with situated learning theory, where knowledge is embedded in social interaction and community practice (Lave and Wenger 1991), helping to explain how participants developed confidence through observation, dialogue and shared experience rather than formal training pathways.

Health professionals demonstrated creativity in filling educational gaps, yet their reliance on experiential learning masked systemic inequities between metropolitan and rural health systems. Participants described the potential value of embedding mental‐health educators or clinical nurse consultants within the ED to provide real‐time specialist support.

The third theme, small‐town mentality, exposes the dual nature of community intimacy, simultaneously protective and burdensome. Familiarity with persons outside the hospital fostered empathy and continuity but also heightened self‐consciousness and stigma. This tension reflects long‐standing evidence that social visibility in rural communities can both strengthen and strain mental health care (Thornicroft et al. 2007; Reed and Fitzgerald 2005).

Participants' deliberate acts of closing curtains, lowering voices and protecting confidentiality reflect what Mol (2008) describes as *care as practice*, evident in how clinicians actively adapted care to preserve privacy and dignity within socially visible communities. These practices highlight the ongoing ethical work involved in navigating care in contexts where anonymity is limited and social relationships extend beyond the clinical setting. For health professionals, this blurred boundary required ongoing ethical vigilance and emotional balance.

In small communities, therapeutic distance is neither possible nor always desirable; instead, clinicians often described balancing familiarity with professional responsibility, maintaining compassion while protecting patients' dignity. This also resonates with Palmer et al. (2019) who highlight the moral and relational dimensions of care, particularly in contexts where professional and community boundaries intersect, as reflected in participants' accounts of navigating dual relationships and maintaining confidentiality within close‐knit communities. Thus, small‐town mentality can be understood not simply as a constraint, but as a site of deeply contextualised, relational ethics.

Viewed together, the three themes represented a coherent picture of rural emergency practice as relationally sustained care under structural strain. Safety, learning and community are intertwined: safety is learned through relationship; education emerges through collective adaptation; and community familiarity shapes both empathy and risk. These interdependencies suggest that improving rural emergency mental health care requires both strengthened system resourcing and support structures, alongside recognition of the relational work clinicians already undertake. Relational practices should not be relied upon to compensate for structural limitations but supported within adequately resourced systems.

The researcher who was employed within the same health service brought an insider perspective to the study. This positionality shaped how participants' accounts were heard and interpreted, particularly in relation to the realities of rural emergency care. Throughout the analytic process, interpretations were discussed with the research team who provided critical distance, challenged assumptions and supported reflexive engagement with the data. This collaborative approach strengthened analytic transparency and depth. Braun and Clarke (2021) argue that transparency of the researcher's influence enhances, rather than diminishes, analytic credibility. Reflexive journaling and dialogue enabled the researcher to acknowledge emotional resonance with participants' experiences of frustration, moral tension and pride in compassionate care. This reflexivity deepened interpretation by revealing how meaning was co‐produced through both participant and researcher perspectives.

## 5 Strengths and Limitations

This study offers important insights into the experiences of health professionals working in a rural emergency department, addressing a gap in the literature where rural voices are often under‐represented. The use of Reflexive Thematic Analysis allowed for a nuanced interpretation of the data and the inclusion of both nursing and medical participants strengthened the breadth of perspectives captured. The researcher's familiarity with the rural context also supported sensitive and informed engagement with participants.

However, the study has several limitations. It was conducted in a single rural emergency department, which may limit the transferability of findings to other settings. The findings are shaped by the specific service configuration of this setting, including limited access to specialist mental health support, reliance on a generalist workforce and resource constraints typical of rural health services. These contextual features likely influenced participants' emphasis on relational approaches to safety, experiential learning and the impact of community familiarity. As such, the findings should be understood as reflecting how mental health care is enacted within this particular rural configuration, rather than representing all rural emergency settings.

As with all qualitative research, the interpretive lens of the first author influenced the analysis, although reflexive processes supported transparency in meaning‐making. The relatively small sample size, while appropriate for in‐depth qualitative inquiry, means the findings should be interpreted as contextually specific rather than generalisable. Despite these limitations, the study offers valuable insights into relational care and safety in rural emergency practice.

## 6 Conclusion

This study explored how health professionals working in a rural emergency department perceive and engage with people presenting with mental illness. The findings suggest that care in rural emergency settings is shaped by structural limitations, relational proximity and the responsibility clinicians assume in the absence of specialist support. Rather than relying solely on formal systems, clinicians negotiate safety, develop skills through experience and respond relationally within resource‐constrained rural contexts. Together, these findings contribute a nuanced understanding of rural emergency mental health practice, highlighting both the adaptive capacities of clinicians and the moral and emotional demands of providing care in settings marked by constraint.

## 7 Relevance for Clinical Practice

This study highlights that mental health care in rural emergency departments is sustained through relational practices rather than formal systems alone. For clinicians, the findings emphasise the importance of teamwork, empathy and contextual awareness when managing safety, learning and stigma in small communities. For services, the study underscores the need for accessible education, embedded mental health expertise and reflective support to reduce moral distress and strengthen workforce capability in rural emergency settings.

## 8 Clinical Implications

Findings from this study emphasise the importance of strengthening relational capacity within rural emergency departments. Embedding mental health expertise through consultation‐liaison or clinical educator roles may provide immediate support, mentorship and guidance to staff managing mental health presentations. Developing accessible and practical education tailored to rural contexts may enhance confidence and competence in assessment and de‐escalation.

Regular reflective supervision and peer‐support forums may support emotional wellbeing, reduce burnout and promote ethical resilience among staff. Strengthening communication and collaboration between EDs and community mental health services may improve continuity of care and recovery‐oriented outcomes. Above all, embedding relational care as a professional capability—rather than an individual attribute—may help support safe, respectful and compassionate care for people presenting with mental illness in rural settings.

## Author Contributions

P.N. conceived the study, conducted data collection, completed data analysis and drafted the manuscript. N.B. and A.H. provided methodological supervision, contributed to interpretation of findings and critically reviewed the manuscript. All authors read and approved the final manuscript.

## Funding

This research did not receive any specific grant from funding agencies in the public, commercial, or not‐for‐profit sectors.

## Ethics Statement

This study received ethical approval from the Darling Downs Health Human Research Ethics Committee (DDHREC/2025/000) and the Monash University Human Research Ethics Committee (MUHREC).

## Conflicts of Interest

The authors declare no conflicts of interest.

## Supporting information


**Appendix S1:** Interview Guide.

## Data Availability

The data that support the findings of this study are available from the corresponding author upon reasonable request.
