# Supplementary material for: How Do Australian Health Professionals Working in a Rural Emergency Department Perceive Their Work With People With Mental Illness? An Interview Study
Source: Int J Ment Health Nurs. 2026 Jul 2;35(4):e70293. doi: 10.1111/inm.70293 (PMC13324965; doi:10.1111/inm.70293)
Supplement: Supplementary file 1 — Appendix S1: Interview Guide. [file INM-35-0-s001.docx]

**Supplementary Appendix 1: Interview Guide**

Q1. Can you describe a situation where you provided care for people with a mental illness in the emergency department? What parts of the situation felt routine, and what parts stood out as different for you?

Q2. What are some of the concerns you have when caring for people with mental health problems?

Q3: Do you think adequate training has been provided to doctors/nurses when triaging people diagnosed with mental health? Is there support available for these doctors/nurses within the ED?

Q4. Could you identify any specific care or support required for people presenting with a mental health crisis in the emergency department?

Q5. Would you like to share your insights and experience towards mental health presentations in this rural emergency department?

Q6. Can you describe any challenges or emotions you experience when caring for people during a mental health crisis in the emergency department?

Q7. How do the challenges of working in a rural emergency department affect the way you approach caring for people with mental health problems?

Q8. If you could make any changes to how mental health care is provided here, what would that be?

Q9. Do you feel that we have covered all the most important topics related to the care of people with mental illness in the emergency department?
